# Translational Geroscience: Emphasizing function to achieve optimal longevity

**DOI:** 10.18632/aging.100694

**Published:** 2014-08-10

**Authors:** Douglas R. Seals, Simon Melov

**Affiliations:** ^1^ Department of Integrative Physiology, University of Colorado Boulder, Boulder, CO 80309, USA; ^2^ Buck Institute for Research on Aging, Novato, CA 94945, USA

**Keywords:** aging, healthspan, lifespan, physiology, biomedical research

## Abstract

Among individuals, biological aging leads to cellular and organismal dysfunction and an increased risk of chronic degenerative diseases and disability. This sequence of events in combination with the projected increases in the number of older adults will result in a worldwide healthcare burden with dire consequences. Superimposed on this setting are the adults now reaching traditional retirement ages--the baby boomers--a group that wishes to remain active, productive and physically and cognitively fit as they grow older. Together, these conditions are producing an unprecedented demand for increased healthspan or what might be termed “optimal longevity”—to live long, but well. To meet this demand, investigators with interests in the biological aspects of aging from model organisms to human epidemiology (population aging) must work together within an interactive process that we describe as *translational geroscience*. An essential goal of this new investigational platform should be the optimization and preservation of *physiological function* throughout the lifespan, including integrative physical and cognitive function, which would serve to increase healthspan, compress morbidity and disability into a shorter period of late-life, and help achieve optimal longevity. To most effectively utilize this new approach, we must rethink how investigators and administrators working at different levels of the translational research continuum communicate and collaborate with each other, how best to train the next generation of scientists in this new field, and how contemporary biological-biomedical aging research should be organized and funded.

## Live Long and Prosper

Spock (Star Trek)

In biology, as in other fields, seemingly intractable problems can provide an opportunity to rethink long-standing scientific approaches. In the case of the rapidly changing demographics of human aging, we face a doozy of a problem: too many older adults in the queue and not nearly enough infrastructure and resources to support their projected healthcare and broader societal needs. Expanding on a recent brief comment [1], in this article we discuss the increasing demand for optimizing health in the context of population aging, the opportunity to achieve this aim using a collaborative translational biological research approach, the importance of assessing physiological function in this new model, and the cooperation, infra-structure, training and resources required for success.

## Optimal Longevity

From a biomedical perspective, the impact of aging on individuals can be viewed in a relatively straightforward manner: development of physiological dysfunction (impairment), which leads to functional limitations (e.g., reduced mobility), increased risk of disease and disability, decreases in productivity, loss of independence, a reduction in quality of life and, ultimately, death. However, when the consequences of biological aging are applied to the large number of aging adults worldwide, economic and social pressures are created on an unprecedented scale [2]. Moreover, apart from the expected burden that societal aging is producing on healthcare systems, government insurance programs and family support networks, comes a new influence: the attitudes of those who are now moving into traditional retirement age. As represented by the baby boomers, i.e., adults born between 1946 and 1964, most of those entering older adulthood today have far different expectations about aging than previous generations [2]. This generation has a strong interest in remaining healthy, active, physically and cognitively fit, productive and independent. They are creating an unprecedented demand for what might be termed ***optimal longevity***—living long, but with an even greater interest in living well.

Investigators working in fields related to the biology and biomedicine of aging (“Geroscientists”) are among those at the forefront for creating solutions to the impending impact of global aging. Several strategies have been identified, the most well-known of which is the “compression of morbidity” paradigm advanced by Fries over 30 years ago [3]. This approach is based on the idea that because most illness today is in the form of chronic diseases, if the onset of these disorders can be delayed to an older age, and the delay is greater than any associated increase in life expectancy, then illness, disability and their sequelae can be restricted to a shorter period at the end of life.

**Figure 1:**
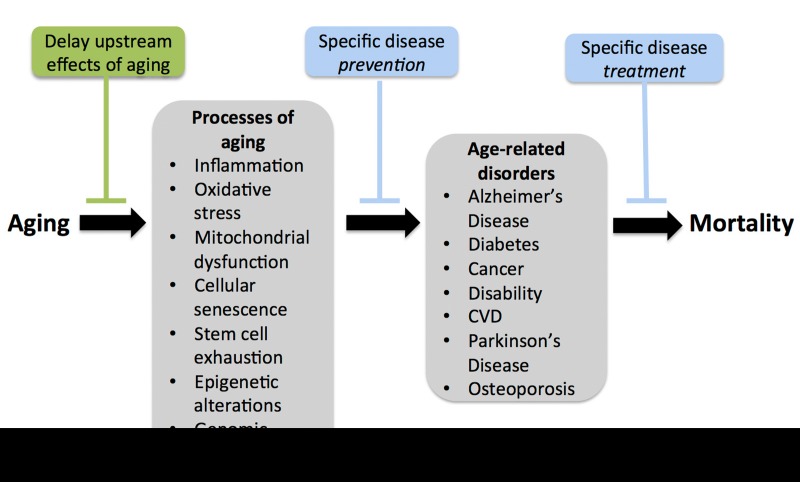
Compressing morbidity by slowing the processes of aging Slowing the fundamental biological processes of aging as a tactic for achieving delaying the age of onset of multiple co-morbidities, as opposed to preventing or treating individual age-associated clinical disorders.

**Figure 2:**
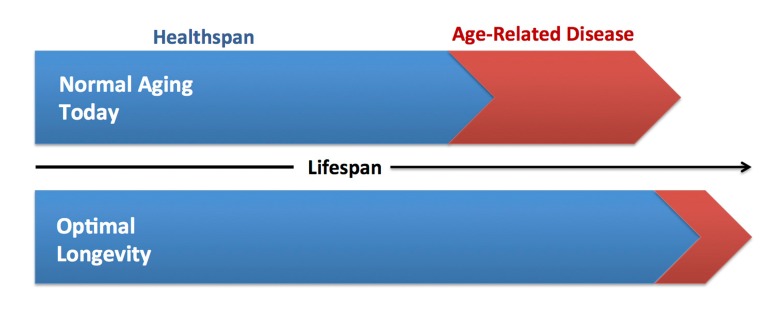
Increasing healthspan and optimal longevity Comparison of current vs. ideal healthspan. Extending healthspan is a critical component of achieving optimal longevity, defined as living long, but with good health, function, productivity and independence.

The key issue is how to best achieve compression of morbidity. Presently there is considerable support for the tactical approach of slowing the fundamental biological processes of aging, as opposed to treating (or even preventing) individual chronic diseases [2, 4-10] (Figure 1). Much of the momentum for this approach has been created by the tremendous advances made over the last 25 years in what is now the routine manipulation of lifespan in model systems such as *Drosophila* or *C. elegans* [11-14]. The idea is that targeting specific ‘upstream’ pathways, originally identified in model systems, holds promise for delaying the age of onset of multiple age-associated comorbidities as a group, whereas delaying the clinical manifestation of a particular disease may simply result in some other age-related disorder “backfilling” the consequent reduction in risk. Slowing aging at the molecular and cellular levels would, theoretically, increase “healthspan”, i.e., the period of life free from serious chronic diseases and disability, thus compressing morbidity and facilitating attainment of optimal longevity (Figure 2).

There is emerging evidence supporting this strategy in animal models in which lifespan has been extended with pharmacological treatment [15-18]. Support also exists in human populations. For example, many centenarians demonstrate delayed clinical manifestation of chronic disease and disability [19, 20], and findings on the California Seventh-Day Adventists are consistent with an increase in both healthspan and life expectancy in that group [21, 22]. Moreover, certain interventions like regular aerobic exercise, appear to increase healthspan as well as survival (mean lifespan), although not necessarily maximal lifespan [23-25]. Ultimately, the degree to which morbidity can be “compressed” with any approach (lifestyle, pharmacological, genetic) will depend on the relative extension of healthspan vs. life expectancy [26].

## Translational Geroscience

Strategies to slow aging and delay age-associated diseases require both an understanding of the basic mechanisms of primary biological aging (potential therapeutic targets) and establishing the efficacy of treatments that favorably modulate (activate or inhibit) those targets. Historically, efforts to investigate such strategies have involved several groups of investigators, including: 1) those studying the basic biological mechanisms of aging and lifespan using model organisms; 2) those investigating the biology of normal aging in groups of healthy adult humans; 3) physician scientists studying older patients in the setting of geriatric medicine; and 4) epidemiologists studying biological aging at the population level. These groups have operated largely in isolation, with their own meetings, investigator networks, experimental approaches and scientific cultures [26].

Recently, however, there has been greater emphasis in adopting a more translational approach in which these groups work collaboratively to facilitate the development of effective treatments to delay aging [4, 5, 26], although this idea had been advanced earlier [8]. Part of this movement has been stimulated by the current interest of some investigators working in the field of basic aging biology to study issues related to healthspan, in addition to their long-standing focus on lifespan. To an extent, the limited studies on healthspan *per se* in model systems such as yeast or *C. elegans* has arisen due to a general paucity of meaningful metrics for assessment. However, with increasing numbers of investigators addressing issues related to healthspan in mice, this situation is rapidly changing. The heightened awareness of the need for interdisciplinary approaches, combined with recent success in the manipulation of lifespan in the model systems, has coincided with the formation of a new interest group in Geroscience, including a recent inaugural meeting discussing many of the key research problems in the field (http://www.geron.org/About%20Us/nih-geroscience-summit).

**Figure 3:**
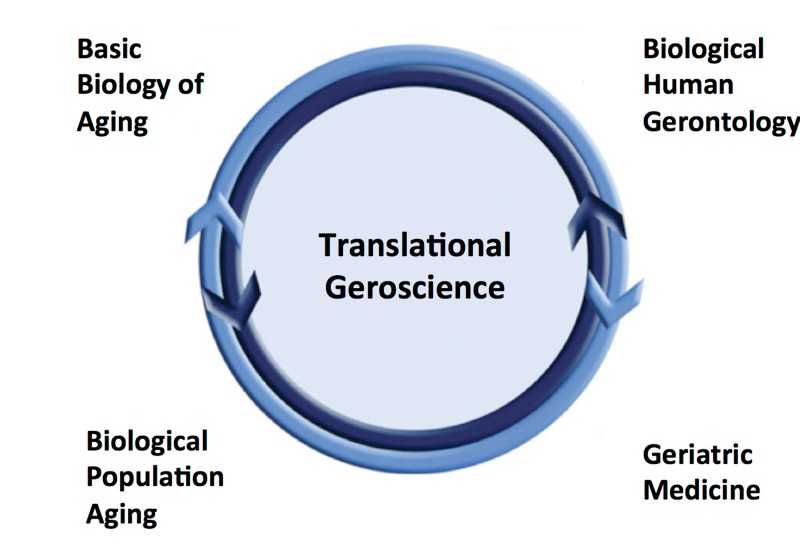
Translational geroscience Translational gero-science represents an integrative model for conducting biological-biomedical aging research leveraging a bi-directional, continuum of observations from basic science to populations using multidisciplinary approaches.

Adopting a more multidisciplinary translational approach to biomedical aging research requires a mutual understanding of the concept of translational research. Some scientists hold the traditional perspective of translational research as a unidirectional process in which original (discovery) observations are made in the basic science laboratory and subsequently extended to humans, often culminating in a clinical trial. In contemporary views, however, translational research refers to a *continuum* in which observations made in preclinical models are first translated to humans in a clinical research setting (bench to bedside) and then to the clinic, eventually resulting in new medical guidelines and public health policy (bedside to community) [27, 28]. Importantly, the translational process is intended to be dynamic and bi-directionaI. Indeed, current translational research constructs emphasize the value of making discovery observations at the clinical or even population level and “reverse translating” to basic science models to discern the underlying mechanisms and identify promising interventions. Extending this concept to biological aging, ***translational geroscience*** can be viewed as a bi-directional continuum that includes investigation from the fundamental mechanisms of aging using basic model organisms to population aging studied in community settings, with the ultimate goal being optimal longevity (Figure 3).

As emphasized in recent perspectives [4, 5, 7, 26], the timing appears to be right for such a movement. The emerging interest of many basic aging biologists in the concept of healthspan has created a new common ground for investigators working at different levels of the translational geroscience continuum. Whereas advances in genetically and molecularly modified animals have revolutionized basic biological research, on the other end, access to a wide variety of samples from human subjects combined with developments in high-throughput molecular analysis (‘omics’) and systems biology now allows novel descriptive and mechanistic observations in populations of humans. This, in turn, provides a platform for conducting translational research using either a forward (bench to bedside to community) or a reverse translational approach. Identifying age-related targets initially in human populations using molecular/systems biological approaches also may provide an experimental advantage for avoiding the false positives often associated with translation of observations from animal models. Indeed, with recent advances in the basic biology of aging there now is a growing list of potential targets and therapeutic agents for delaying aging that are awaiting translation to humans [4, 5, 26, 29], and new evidence suggests that strategies effective in slowing aging likely will result in significant health and economic benefits to society [6]. In addition to the healthcare-driven economic demands on governments and insurance programs, there is increasing public interest in healthspan-promoting solutions, and this influence, in itself, is creating academic research and commercial opportunities aimed at addressing that demand.

## Healthspan and Function

**Figure 4:**
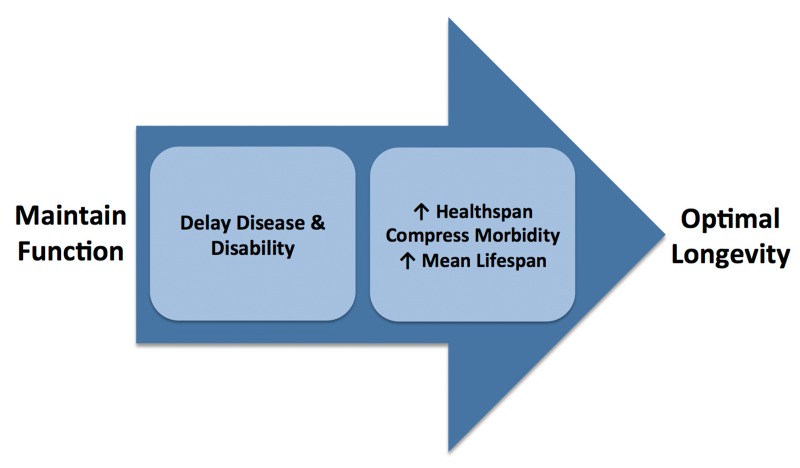
Role of preserved function in achieving optimal longevity Maintenance of good physiological function with aging delays the onset of chronic diseases and disability, increases healthspan, compresses morbidity, extends mean lifespan and helps attain optimal longevity.

An important question is how to best approach future research aimed at increasing healthspan using a translational geroscience platform. In this context, it is helpful to emphasize that healthspan is more than simply freedom from major diseases. Good physical and cognitive function that allows sufficient physiological reserve to effectively interact with our environment and perform the activities of daily living is an inherent component of any “healthy” period of life. One could be free of major clinical diseases, yet experience a shortened healthspan because of functional limitations due to sarcopenia or other subclinical effects of primary aging. Moreover, physiological dysfunction is a major gateway to increased risk of future age-related chronic diseases, disability and, therefore, reduced healthspan [30-33]. Impaired function also is a well-established independent predictor of mortality with aging in both preclinical models and human populations [34-37]. As such, a key goal for increasing healthspan and achieving optimal longevity must be the preservation of physiological function at the highest possible levels with advancing age (Figure 4).

The importance of function and its assessment form much of the basis of past and current research in human gerontology and geriatric medicine. Although function has been measured in some studies employing mammalian and non-mammalian models of aging, this is far less consistently the case compared with human investigations [26]. Rather, most basic research in aging to date has focused on modifying a molecular target that is suspected of having a functional influence, and/or assessing biochemical and histopathological changes within the animals being subjected to intervention. However, genetic or pharmacological manipulation of molecular signaling pathways does not, by itself, ensure that function will be altered because of physiological redundancy [38], even under conditions in which some other phenotypic change has been induced, including lifespan extension. To facilitate translation to humans it is critical that future studies of the fundamental biological mechanisms of aging characterize the *functional effects* of the molecular and cellular pathways under investigation [8, 26].

**Figure 5:**
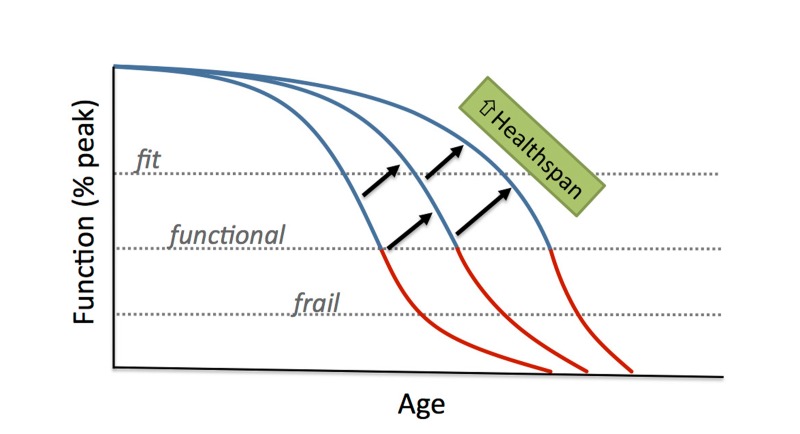
Compression of physiological dysfunction with aging Compression of physiological dysfunction (or rectan-gularization of function) with aging would allow function to be well maintained with advancing age, limiting the occurrence of major functional limitations to a period near the end of life, thus enhancing healthspan.

By determining the mechanisms responsible for functional declines with aging and establishing interventions that act to preserve function by suppressing those mechanisms, it may be possible to slow or delay the loss of physiological function with advancing age, thus restricting major functional limitations to a shorter period at the end of life (“compression of dysfunction” or “rectangularization of function” with aging). This would, in turn, serve as a major mechanism for achieving extension of healthspan (Figure 5). In the never-ending search for biomarkers of aging and healthspan, much of the focus has been placed on novel molecular signatures. Physiological function represents a set of already established, straightforward and practical evidence-based biomarkers that can be utilized to assess the biological effects of aging and the effectiveness of treatments.

## Translational Assessments of Function

Presently there are a number of *in vivo* and *ex vivo* translational models and techniques to assess function, its underlying mechanisms, and responses to treatments with aging [27]. Several of these approaches allow the same functions to be studied in preclinical models and humans, with the potential to extend observations to population aging [27, 39-42]. Recent development of animal models for assessing declines in integrative assessments of motor and cognitive function with primary aging, as well as syndromes of aging such as frailty, provide previously unavailable experimental opportunities for determining the efficacy of therapies on functional outcomes [40, 41, 43-46]. Such models open possibilities for more direct translation of interventions from model organisms and rodents to populations of middle-aged/older adult humans using new instruments like the NIH Toolbox test batteries for assessment of motor, cognitive and sensory function [47].

Despite this growing number of tools, as summarized in Table 1, much work lies ahead to fully tap the potential insight provided by assessments of function in basic aging research. Existing measurements of function currently are underutilized in rodents and other model organisms. To initiate change in this area, we must start with greater recognition of the need to assess function in basic studies of aging as essential biomarkers of healthspan, as has historically been the case in human aging research. We then must begin to utilize existing measurements of function in animal models of aging, particularly those assessments that provide translational insight into function in humans. While integrating presently available methods, we must initiate an aggressive effort to develop new, clinically relevant assessments of function and supporting mechanistic techniques for use in model organisms of aging.

**Table 1:** Action items for incorporating assessments of function into animal studies of aging

1	Recognize the importance of functional assessments in model organisms
2	Commence utilizing currently available functional measurements
3	Establish new methods directly applicable to human studies
4	Determine limits of precision, and robustness of functional assays
5	Perform serial assessments of function over time with age or treatment
6	Report inter-individual variability in function with age/treatment
7	Characterize multiple tissue, organ and integrative (e.g., motor) functions
8	Assess function under baseline and other human-relevant conditions
9	Emphasize standardized, cost-effective & non-proprietary methods

In designing new approaches for assessing physiological function with aging and/or healthspan-promoting treatments in animal models, several features should be considered. Developing non-invasive assessments of function using imaging, behavioral measurements, and other techniques that can be performed serially in animals over time would provide novel insight into temporal patterns of functional decline with aging that would parallel longitudinal studies in humans. Such approaches could be used under control conditions to establish the functional effects of primary aging in wild type vs. genetically modified animals, and also in response to promising interventions for preserving function with aging. Developing functional assessments on outcomes that are directly translatable to those obtained in ongoing longitudinal studies in humans such as the Dynamics of Health, Aging and Body Composition (Health ABC) Study would be particularly insightful. As in current studies of human aging, future approaches in animal models must incorporate assessments of function in multiple tissues and organs, as well as measurements of integrative motor (strength, endurance, locomotion, etc.) and cognitive functions. Indeed, modeling of assessments of “functional status” in humans that are most likely to be approved by regulatory authorities for future clinical trials of healthspan-extending pharmaceutical agents in at-risk older adults should be a key goal [37, 40, 41, 48, 49].

Other methodological properties also should be emphasized in assessments of function in animal models of aging. To facilitate comparisons among studies, measurements should be reliable, highly standardized and, to help ensure translational relevance, validated against human populations whenever possible [40, 41, 43]. To provide the greatest possible access for investigators, assessments of function also should be cost-effective and non-proprietary, as in the case of the recently launched NIH Toolbox testing batteries in human subjects [47]. Function should be assessed in model organisms under similar conditions as in human studies, i.e., under resting conditions and, to test functional reserve capacity, in response to real-life physiological perturbations such as physical exercise, cognitive tasks, and immunological challenge. Although it is important to characterize stress resistance, the historical use of non-physiological conditions to assess these properties in animal models of aging has, in many cases, little relevance to humans.

Finally, assessing inter-individual differences in function has been a common feature of investigations in human studies, providing important insight into factors that influence function with aging or interventions. In contrast, reporting of inter-animal differences in biological variables is rare in basic studies of aging, and needs to be incorporated into future investigations with functional outcomes.

Emerging technologies are providing opportunities for complementary analyses of tissues from which to assess and interpret functional outcomes in animal models of aging. These include newly developed methods for non-invasively measuring total muscle mass in mammals [22, 49]. When applied to rodent models of aging, these assessments allow investigators to determine if genetic manipulations or pharmacological interventions delay loss of skeletal muscle mass with advancing age, a high priority in biomedical aging research. Such measurements of muscle mass, along with corresponding assessments of muscle strength, will allow basic scientists in the biology of aging to more effectively study sarcopenia in animal models in parallel with newly established clinical guidelines [46, 50]. Indeed, sarcopenia is an example of a major geriatric problem that has been studied largely in isolation in the basic and clinical aging communities, but is most effectively addressed with interdisciplinary, translational assessments of structure-function [51]. Another area in which such approaches can be applied is high resolution imaging of bone in aging cohorts of animals using microCT. Technical advances in this field now permit serial quantification of several different clinically relevant metrics with direct implications for age-associated bone remodeling in humans [11, 48, 50, 52]. Serial (non-terminal) biopsies of tissues such as skeletal muscle could provide corresponding information on the molecular and biochemical changes mediating the effects aging and interventions on function. Table 2 shows examples of functional and supporting structural measurements available in both rodents and human subjects.

**Table 2:** Examples of functional and function-supporting measurements available in rodents and humans.

Common Functional or Biomarker Measures	Rodents	Humans
Aerobic exercise capacity (VO_2_max)*	✔	✔
Autonomic nervous system (HRV; SNS activity)	✔	✔
Body composition (lean and fat mass; bone density; DXA, CT, MRI)	✔	✔
Body temperature	✔	✔
Cardiovascular (BP; pulse wave velocity; endothelial function; cardiac-echocardiography)*	✔	✔
Cognitive function (executive function; memory; etc.)*	✔	✔
Energy expenditure (metabolic rate); physical activity*	✔	✔
Glucose tolerance; insulin sensitivity	✔	✔
Inflammation/oxidative stress (superoxide production; cytokines; antioxidants)	✔	✔
Kidney function (GFR; BUN; urinary protein)	✔	✔
Motor/physical function (strength; endurance; balance; coordination)*	✔	✔
Pulmonary function	✔	✔
“Omics” (tissue biopsies; blood cells; platelets; plasma; serum; feces; saliva; etc.)	✔	✔

HRV, heart rate variability; SNS, sympathetic nervous system; DXA, dual x-ray absorptiometry; CT, computed tomography; MRI, magnetic resonance imaging; BP blood pressure; GFR, glomerular filtration rate; BUN, blood urea nitrogen; * has been at least indirectly assessed in invertebrate models of aging

Lastly, it is important to stress that physiological function can be enhanced during aging not only during lifelong interventions such as caloric restriction or voluntary wheel running [53-56], but also in response to treatments initiated later in life [15, 39, 42, 46, 57]. Later-life interventions showing efficacy for improving function in basic models of aging can be tested in healthy middle-aged/older adults with baseline dysfunction and who, therefore, are at increased risk of disease and/or disability, as well as in patients with existing age-related disorders [4, 8, 42]. Whatever the approach or target population, determining the efficacy of novel treatments for optimizing physiological function with aging is one of the most important frontiers in biomedical aging research.

## Infrastructure and Resources

The ultimate success of translational geroscience research, including efforts aimed at optimizing function with aging, will depend in large part on overcoming present limitations regarding infrastructure, resources, methods of communication and collaboration, and scientific training opportunities and approaches. As emphasized in earlier perspectives [4, 5, 26], there are a number of opportunities, and challenges, for translating promising interventions and therapies from the lab to the clinic and communities.

### Translation of Function-Enhancing Treatments: Options and Obstacles

In the area of promising dietary and pharmacological strategies, the National Institute on Aging (NIA) Interventions Testing Program (ITP) program has become a highly successful source of potential treatments to reduce age-associated pathologies and extend lifespan in mice. Moreover, independent laboratories working in basic aging biology recently have produced a remarkable number of potential targets and associated target-modulating treatments worthy of translational consideration [4, 5, 26, 29]. Overall, it seems likely that identification of candidate therapies from preclinical models will *not* be the major limitation for establishing effective interventions to slow the effects of aging and delay the onset of age-associated co-morbidities. However, to identify the most promising targets and agents, we must provide investigators working in basic aging research the training and access to resources necessary to broadly assess function in their models. This capacity has been established in selective independent laboratories, as well as in phenotyping core laboratories at specific institutions (the Healthspan Assessment Laboratory at the Mayo Clinic in Rochester, Minnesota is a good example). Nevertheless, greater development of the infrastructure required for measuring functional outcomes in model organisms will be necessary to ensure that the interventions leaving the basic aging research pipeline have the strongest possible potential for enhancing healthspan in humans.

One of the main obstacles for translation of treatments to improve function with aging lies in the initial steps from assessments in animal models to testing for safety and efficacy in human subjects (phase I and II clinical trials). The process for bringing new prescription agents targeting aging to market has been described in detail by Kirkland [4], and the steps, time lines and costs involved are extensive. However, development of drugs for older patients with geriatric syndromes such as frailty, as well as clinical disorders that are antecedents of these syndromes, clearly is an important goal and area of interest for the pharmaceutical industry.

**Figure 6:**
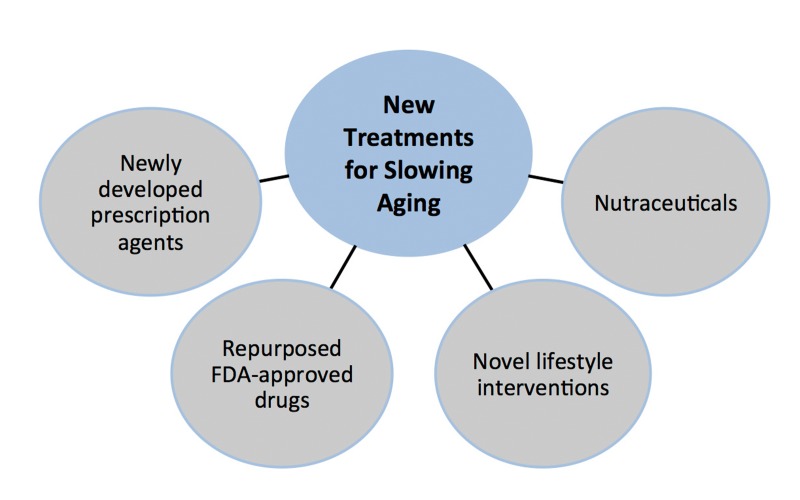
Sources of new treatments for slowing effects of aging Sources of new treatments for slowing the fundamental processes of aging include newly developed prescription agents, repurposed FDA approved drugs, novel lifestyle behaviors (e.g., intermittent fasting) and nutraceuticals (dietary supplements, medical foods and functional foods).

Complementary options to new proprietary prescription drug development also exist, and may represent, in some cases, a nearer-term source for new therapies with function-enhancing effects for older adults (Figure 6). For example, widely used FDA approved drugs with established safety and efficacy for treating age-associated clinical disorders such as cardio-metabolic diseases (e.g., metformin, statins, renin-angiotensin system inhibitors, recent generation beta-blockers) could undergo repurposing for treatment of at risk older adults or patients with aging syndromes. Although such agents could be prescribed presently for their off-label effects in cases in which the existing evidence supports likely efficacy, broad use of these drugs likely will require new trials with appropriate subject groups and clinical endpoints recognized by drug regulatory authorities.

Nutraceuticals (nutriceuticals) are another category of pharmacological agents that may hold promise for preserving physiological function with aging. These are foods or food supplements with naturally occurring ingredients purported to have some type of health benefit, and include dietary supplements, functional foods and medical foods. Resveratrol, antioxidant vitamins and polyphenols, anti-inflammatory compounds such as curcumin, inorganic nitrites/nitrates, omega 3 fatty acids and vitamin D are a few examples of the hundreds of existing nutraceuticals with purported health-promoting effects for aging. These compounds may be more cost effective as healthspan-enhancing pharmacological options, but informed use presently is limited by lack of evidence for efficacy and other problems [42].

Healthy lifestyle behaviors presently are the most well established healthspan-enhancing strategies. In this context, lifestyle interventions involving “stimulus-varying” forms of physical exercise (e.g., low-high interval training), novel, but feasible dietary modification (e.g., intermittent fasting/time restricted feeding; healthier diet composition), and behavioral stress reduction therapies are among the many non-pharmacological, readily testable strategies for slowing primary aging, enhancing physiological function and preventing/delaying chronic age-related diseases. Continued study of such interventions is important given that many pharmacological treatments presently under development are based on mimicking the biological actions of healthy lifestyle behaviors.

An important question concerning these therapeutic options is how basic scientists who are interested in translating their preclinically-tested treatments can do so given the equipment, facilities, personnel, experimental skills and knowledge of regulatory processes needed to conduct initial studies in humans. Moreover, clinical investigators who have the experience and infrastructure to conduct trials on older adults may not have the resources to test compounds identified from basic aging research. For trials involving FDA-approved agents, nutraceuticals not requiring FDA approval, and/or lifestyle interventions, an ITP-like program in which such putative therapies could undergo near-term testing for safety and efficacy in humans might be helpful (Translational Testing Program--TTP). As with the ITP, such a program might involve simultaneous testing of a particular treatment in 2 or 3 cooperating centers to help ensure the validity of any effect observed. Academic institutions with investigators experienced in conducting interventions in middle-aged/older populations and with the appropriate clinical research infrastructure would represent potential assessment sites. In the U.S., many medical schools have NIH-supported Clinical and Translational Research Centers that could serve as testing facilities, and similar clinical research centers exist throughout Europe and other countries with modern biomedical research infrastructures. The preclinical-to-clinical research programs created by the National Cancer Institute in the U.S. to facilitate translation of novel cancer therapies has been advanced as another possible model to fast-track promising treatments targeting aging [4].

### Collaboration, Funding and Research Training

To more fully cultivate translational geroscience and the steps towards achieving optimal longevity, we must reconsider the ways in which investigators and organizations with interest in the biology and biomedicine of aging interact and communicate, how future generations of scientists in the field should be trained, and how aging research should be funded [4, 5, 7, 9, 26].

Despite much-needed recent efforts, organizers of scientific meetings and workshops historically devoted to the basic biology of aging must continue to be proactive about integrating sessions and investigators working in translational biological gerontology, geriatric medicine and epidemiology, and vice-versa. In these settings, the greatest progress is achieved when basic scientists with their knowledge of the mechanisms of aging, potential molecular therapeutic pathways, and novel treatment compounds interact directly with investigators skilled in the physiology, cognitive neuroscience and epidemiology of human aging, as well as with physician-scientists who have the first-hand knowledge of geriatric medicine to identify the most pressing problems in aging. The most effective solutions for these problems will *not* be established by continuing to work in isolation, and by simply paying lip service to the need for greater multidisciplinary interactions. Cross-disciplinary white papers, guidelines, funding opportunities and other publications should be among the useful products created by these interactions.

Investigators and funding organizations need to work together to create new, properly incentivized funding opportunities and training programs to support translational geroscience research and training. Such a portfolio should include individual investigator awards, multi-investigator project grants, and research career development awards. Individual investigator awards might emphasize the development of translational experimental designs and methods within the laboratory's focus and expertise. Multi-investigator translational grant mechanisms might require interdisciplinary teams to be formed to bring each of their unique investigative skills to bear on thematic issues of particular importance in biomedical aging research. Individual research training awards and institutional training grant programs, both pre- and post-doctoral, could involve mentoring networks of experienced investigators working at various levels of observation from basic to population aging biology. Via rotations or other formal experiences, trainees would be exposed to research being conducted at various stages of translation research in order to understand the full scope of the efforts involved, as well as the strengths and limitations associated with each level of observation and associated experimental approaches. Further development of undergraduate and graduate degree programs offering academic and research training in translational geroscience should be encouraged. We also must consider if the current organization of funding bodies in which basic and clinical research related to the biological aspects of aging are administered in separate programs, is the most effective format for advancing this new model of biomedical aging research.

Finally, new sources of funding will be needed for timely advancement of potential healthspan-extending interventions. In the U.S., government biomedical research budget restrictions preclude the ability to fully support the explosion of potential therapeutic targets and compounds presently being identified by investigators working in the basic biology of aging, nor the translation of those exciting observations to aging in humans. Additional charitable foundations with the necessary interest and resources (a “Gates Foundation for Aging”?), contributions from venture philanthro-pists, pharmaceutical companies developing new treatments for aging, contributions from large insurance companies and other industry stakeholders in promoting healthy aging, wealthy private donors, direct public fundraising and other sources will be needed to properly support an ambitious program of contemporary translational biological aging research. Parenthetically, will be interesting to observe developments in the biotechnology industry in this regard. Although there have been several high profile attempts over the last 20 years or so to develop commercial biotechnologies for moving discoveries in the biology of aging into the clinic (Geron, Elixir, Sirtris, etc.), none have succeeded in their initial goals. It will be especially intriguing to observe this space expand, and the recent entry of companies with substantial financial backing such as Calico (Google) will be worth monitoring.

**Figure 7:**
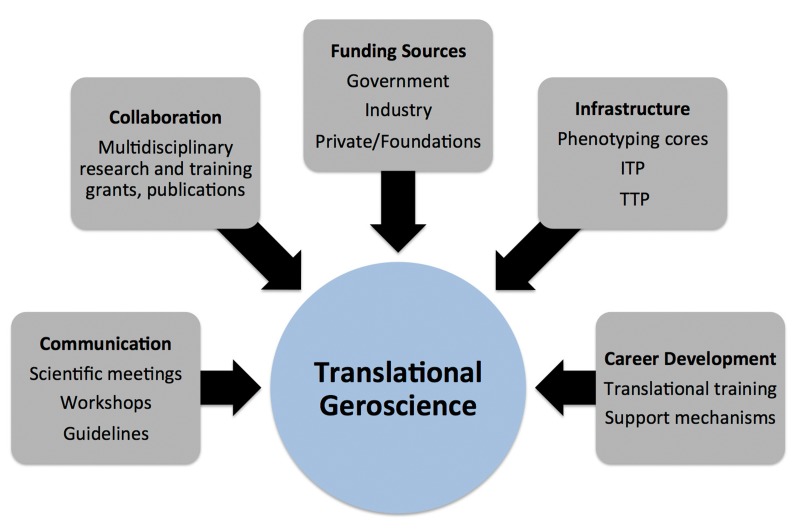
Key components of translational geroscience Essential elements required for successfully conducting future translational geroscience research include effective communication, collaboration, funding sources, infrastructure and career development mechanisms. ITP, Interventions Testing Program; TTP, Translational Testing Program.

Overall, we must establish a new “big tent” culture that bridges the biology of basic aging research, human gerontology, geriatric medicine and population aging based on effective, properly coordinated mechanisms of communication, collaboration, experimental science, infrastructure, research and academic training, and extramural funding (Figure 7).

## Conclusions

The inevitable biological process of age-related physiological dysfunction leading to increased risk of chronic disease, disability and associated socio-economic consequences, when combined the rapidly changing demographics of aging and new attitudes about growing older, are creating an unprecedented demand for optimal longevity--living long, but with increased healthspan (wellness-fitness). To meet this demand, investigators studying aging from model organisms to populations of older adults must work collaboratively at a level far greater than in the past, using a highly integrative approach that could be termed translational geroscience. A major focus of this approach should be on optimizing physical and cognitive function throughout the lifespan, as this is a key trigger for morbidity and disability with advancing age. To properly support translational geroscience research, we must reconsider and, to some extent, reinvent how investigators throughout the biological aging research continuum communicate and interact scientifically, and are trained and funded. These changes must be made now as the initial wave of baby boomers already has reached traditional retirement age, and we are far from having the necessary intervention strategies implemented and infrastructure developed to meet their basic healthcare needs and expectations for optimal longevity.
